# MMBGR protocol – diagnostic accuracy of clinical examination in infants

**DOI:** 10.1590/2317-1782/e20240315en

**Published:** 2025-12-08

**Authors:** Anna Luiza dos Santos Matos, Giédre Berretin-Felix, Íkaro Daniel de Carvalho Barreto, Ricardo Queiroz Gurgel, Andréa Monteiro Correia Medeiros

**Affiliations:** 1 Programa de Pós-graduação em Ciências da Saúde, Universidade Federal de Sergipe – UFS - Aracaju (SE), Brasil.; 2 Departamento de Fonoaudiologia, Faculdade de Odontologia de Bauru, Universidade de São Paulo – USP - São Paulo (SP), Brasil.; 3 Centro Brasileiro de Pesquisa em Avaliação e Seleção e de Promoção de Eventos – CEBRASPE - Brasília (DF), Brasil.; 4 Departamento de Fonoaudiologia, Universidade Federal de Sergipe – UFS - São Cristóvão (SE), Brasil.

**Keywords:** Speech, Language and Hearing Sciences, Infant, Clinical Diagnosis, Validation Study, Sensitivity and Specificity, Myofunctional Therapy, Stomatognathic System

## Abstract

**Purpose:**

To present the diagnostic accuracy of the clinical examination with scores, the Orofacial Myofunctional Evaluation Protocol MMBGR for Infants and Preschoolers, for the age group of six to 23 months.

**Methods:**

Diagnostic accuracy validation using a convenience sample. Images from 76 participants were analyzed by groups of three speech-language pathologists, who individually and separately assessed the images, with agreement between two of them considered valid. An electronic form was used to assess the domains of the orofacial myofunctional evaluation, Orofacial Myofunctional Disorder (OMD), and the need for referrals. The responses of speech-language pathologists who provided opinions based on clinical experience, without using the protocol (gold standard), were compared with those provided by speech-language pathologists who used the protocol (index test). The Receiver Operating Characteristic Curve (ROC) method was employed, and cut-off points were assigned using the R Core Team (2022) software, yielding sensitivity and specificity values.

**Results:**

For infants aged six to 11 months, diagnostic accuracy was not ideal for orofacial structures, orofacial functions, and Orofacial Myofunctional Disorder (OMD), but reasonable for tone (70%). For infants aged 12 to 23 months, accuracy was reasonable for most domains— orofacial structures, orofacial functions, tone—and ideal for OMD (88.9%). The OMD cut-off point is 9 (for ages 6 to 11 months) and 14 (for ages 12 to 23 months). Accuracy was reasonable for multidisciplinary referrals but not ideal for speech-language pathology referrals.

**Conclusion:**

The Orofacial Myofunctional Evaluation Protocol MMBGR for Infants and Preschoolers, when analyzed through photos and videos, is accurate for assessing infants aged six to 11 months only in terms of tone; and for infants aged 12 to 23 months, it is accurate across all exam domains, being ideal for diagnosing OMD. The instrument is reasonable for determining the need for multidisciplinary referrals in infants.

## INTRODUCTION

The expansion of Speech-Language Pathology has generated a need for scientific and technological advances, including the field of Orofacial Motricity (OM). This area is responsible for the promotion, prevention, diagnosis, and rehabilitation of orofacial structures and their functions, specifically breathing, sucking, chewing, swallowing, and speech^(1)^. Along with the expansion of the field, the demand for timely diagnoses has become increasingly emphasized, particularly in the case of children. Early assessment can provide essential support for rehabilitation and referrals during the initial stages of life, as well as for organizing services, support systems, and resources, thereby preventing greater difficulties in later stages^(2)^.

With the aim of standardizing speech-language assessment, especially in cases that require interventions during childhood, the Federal Council of Speech-Language Pathology (CFFa), through Resolution No. 414, established the recommendation for the use of instruments in clinical practice for the diagnosis and treatment of communication disorders, which include protocols, tests, software, and other resources^(3)^. Currently, there are already some recognized protocols for the infant age group (from 1 to 23 months of life), with certain validation phases already conducted, such as the OMES-E Infants protocol^(4)^ and the MMBGR – Infants and Preschoolers protocol l^(5-7)^.

A validated instrument enables the reliable and accurate recording of data, allowing for a detailed analysis of results that can contribute to patient care, facilitate case discussions among different healthcare professionals, and serve as a standardized document for individual assessment^(7,8)^. Despite its great importance, the number of assessment protocols in OM clinics focused on pediatrics remains limited, which underscores the need to expand research aimed at developing and validating instruments for early ages.

The lack of speech-language assessment and/or intervention may lead to consequences that persist into adulthood, such as respiratory, feeding, and communication difficulties^(9)^. In addition, given the relationship of Speech-Language Pathology with other health fields, such as Medicine, Dentistry, Physical Therapy, Occupational Therapy, and Psychology, professionals must be able to contribute to systematic, rigorous, and comprehensive assessments, especially in cases of multidisciplinary care^(10)^.

The potential of the MMBGR – Infants and Preschoolers protocol is noteworthy in recording characteristics considered as alterations, particularly orofacial myofunctional ones, in order to standardize assessment, guide therapeutic planning, and serve as documentation for multidisciplinary practice and referrals^(5,6)^. This protocol was published, presenting the stages of content validation, response processes, and reliability; however, to date, the stage of diagnostic accuracy validity, which involves defining measures of diagnosis, has not been carried out^(11)^, and this also contributes to referral decisions.

Diagnostic accuracy measures how well a given test can correctly identify individuals with alterations and exclude those without them. The result of the intended test (index test) must be compared with that of a test considered the reference standard (gold standard). The comparison between these tests should indicate predictability, referring to the test’s ability to anticipate outcomes, which encompasses sensitivity, specificity, and predictive values^(12,13)^.

The present study presents the phase of diagnostic accuracy validation for the infant age group of the MMBGR – Infants and Preschoolers protocol, specifically the Orofacial Myofunctional Clinical Examination, including scores, as well as the need for speech-language and/or related referrals.

## METHODS

This is a validation study regarding diagnostic accuracy, designed prospectively and based on convenience sampling, conducted with the authors' consent. It was approved by the Research Ethics Committee (REC) of the Federal University of Sergipe, under opinion number 5.147.320, and comprises the description of the diagnostic accuracy validation phase of the Orofacial Myofunctional Clinical Examination Protocol – MMBGR – Infants and Preschoolers^(5)^, referring to the age range of 6 to 23 months.

To understand the diagnostic accuracy validation phase presented here, it is necessary to note that it is related to the validation stages conducted in previous years, as part of a postdoctoral activity carried out by the research coordinator/supervisor. At that time, consent was obtained from the institutions and from the parents or guardians of the infants through the prior signing of the ICF.

Speech-language pathologists were recruited based on the analysis of their Lattes curricula, according to the study’s inclusion criteria, which required the professional to be a “specialist in OM.” Specialists were identified through the Federal Council of Speech-Language Pathology (CFFa) website, covering different regions of the country. Following this survey, each professional was contacted and invited to participate in the project by the principal investigator through prior contact via instant messaging applications and electronic mail (email). Those who expressed interest received, by email, the materials containing the ICF and a link for its printing, as recommended by the REC.

Additionally, via email, a link was sent to the professional characterization form, where consent to participate in the study had to be indicated, along with another link corresponding to the completion of the evaluation related to the clinical cases to be analyzed. A cloud folder containing static and dynamic images of the infants to be assessed was also shared.

### Previous stages

The MMBGR – Infants and Preschoolers protocol had already undergone two validation stages: content validity of the instrument and validity based on response processes through the administration of the clinical examination and assessment of test reliability^(5,6)^.

At the time of the clinical examination, a database was created with static and dynamic images of infants attending institutions (a university hospital and daycare centers) in the states of São Paulo and Sergipe, who met the inclusion criteria of not presenting speech-language complaints, neurological diagnoses, or developmental disorders^(5)^.

### Composition of participant groups (infants)

The participants’ images were divided into two groups based on age range: G1, corresponding to infants aged 6 to 11 months and 29 days, with 40 cases; and G2, corresponding to infants aged 12 to 23 months and 29 days, with 36 cases.

The distribution of the groups by age range followed the same standardization as the original protocol^(5)^, including the items and domains to be assessed by age. Thus, all items analyzed were common to both age groups, except for chewing, which in the 12- to 23-month group was evaluated with the introduction of solid food consistency, a procedure not applied to the younger group. The authors consider that there is a gradual process of change in food acceptance, with the exploration of different textures and flavors occurring between 12 and 24 months^(14)^.

Regarding the domains mentioned, the extraoral examination included items related to the face, lips, and mandible; the intraoral examination focused on the mucosa of the lips, cheeks, tongue, palate, palatine tonsils, and teeth. An evaluation of the data related to the tone of the lips, tongue, chin region, and cheeks was also conducted. For the analysis of stomatognathic functions, dynamic images of breathing, sucking, swallowing of liquids, semi-solids, and solids, as well as chewing, were provided. In addition to the analyses involving orofacial myofunctional domains, at the end of the form, the evaluator was required to indicate, in the general domain, whether the assessed participant presented with OM disorders, whether there was any difficulty in completing the form, and whether speech-language and/or multidisciplinary referrals (Dentistry, Otorhinolaryngology, among others) were necessary.

Chart 1 describes the proposed analysis by the speech-language pathologist, categorized by the infant's age group.

**Chart 1:** Items evaluated by speech-language pathologists, by infant age group

Age Group	Items Evaluated
06-11 months	✔ Orofacial Structures (intraoral and extraoral)
	✔ Tonus
	⮚ Breathing
	⮚ Sucking/Swallowing (bottle or breast)
	⮚ Swallowing of semi-solid food (porridge, purée, or mashed food)
12-23 months	✔ Orofacial Structures (intraoral and extraoral)
	✔ Tone
	⮚ Breathing
	⮚ Sucking (bottle or breast)
	⮚ Chewing: solid/semi-solid
	⮚ Swallowing: semi-solid (porridge, purée, mashed food); solid or semi-solid (food cut into very small, soft pieces or shredded); and liquid

### Diagnostic Accuracy: index test and gold standard test

#### Index test

In the previous stage, regarding validity based on response processes, with the assessment of test reliability^(5,6)^, calibrated speech-language pathologists evaluated the images obtained from the infants using the MMBGR – Infants and Preschoolers protocol, with inter- and intra-rater agreement being verified^(5)^ for each item of the instrument. These evaluations (stored in the researcher’s database) were considered in the present study as the “index test”.

#### Gold Standard Test

Speech-language pathologists specialized in OM, with clinical experience in infants and who had not participated in the previous validation stages of the MMBGR – Infants and Preschoolers protocol, composed the analysis defined as the “gold standard test.” In total, eight specialists agreed to participate and formed the “committee of specialist speech-language pathologists (evaluators)” of the gold standard group. They completed their professional characterization and conducted the image analyses individually and separately, without using the MMBGR – Infants and Preschoolers protocol.

The evaluators responded to two electronic forms. The first, a Professional Characterization form, contained questions on age group, region of practice, academic degree, teaching activity, years of experience, and clinical work with infants. The second referred to the orofacial myofunctional clinical examination, designed to include the evaluation of responses in cases, considering both specific and general domains. It was established that each infant in groups G1 and G2 would be evaluated by a trio of specialists.

In the orofacial myofunctional evaluation, the gold standard group of speech-language pathologists assessed the same infant images (database) without using the MMBGR – Infants and Preschoolers protocol, relying instead on their professional experience in OM. They were asked: Within the presented domain, were alterations identified? Yes or no. In the gold standard group, no scores were assigned; the evaluator simply indicated whether or not an alteration was present.

The following items were assessed.

Domains:

Extraoral examination (face, lips, and mandible);

Intraoral examination (lips, cheeks, tongue, palate, palatine tonsils, teeth, and occlusion);

Tone (lips, chin region, tongue, and cheeks);

Orofacial functions – G1 (breathing, sucking, and swallowing of semi-solid food);

Orofacial functions – G2 (breathing, sucking, chewing, swallowing of liquid, semi-solid, solid, and pasty food).

General aspects:

Does this child present with Orofacial Motricity (OM) alteration?

Does this child require speech-language therapy?

Is there a need for referrals? (otorhinolaryngology, dentistry, others)

Regarding completion:

Did you encounter any problems filling out this form?

The specialists’ responses for each clinical case were entered into the electronic forms provided. They were tabulated and stored on a digital platform, analyzed within groups, and agreement was expected regarding the presence or absence of OM alteration from at least two of the three specialists who analyzed each case.

### Analyses for Diagnostic Accuracy Validation

The proposal was to compare the results of the “gold standard test” with those of the “index test.” Thus, the aim was to define how the evaluation process using the Orofacial Myofunctional Evaluation Protocol MMBGR – Infants and Preschoolers (index test) was effective when compared to clinical evaluation without the use of the MMBGR – Infants and Preschoolers protocol (gold standard test), and whether it would be able to predict it.

For the diagnostic accuracy analysis to be validated, it was necessary to specify predictability. Therefore, the Receiver Operating Characteristic (ROC) curve method was used^(13)^, since, through it, the area under the curve can be recognized, in addition to making it possible to determine sensitive cutoff points for alterations in the items evaluated in the Orofacial Myofunctional Evaluation Protocol MMBGR – Infants and Preschoolers.

With the detection of each cutoff point, sensitivity and specificity values were also calculated. It is worth noting that there may be no relationship between OM alteration and the scores of the Orofacial Myofunctional Evaluation Protocol MMBGR – Infants and Preschoolers, by domain (AUC = 0.5), through the Z test for proportions^(15)^. Regarding the analysis, in the area under the curve (AUC), the probability of identifying individuals who are true positives for OM disorders and those who are not becomes greater. An optimal test will have an AUC value close to 1.0. Therefore, the closer to 1.0, the higher the sensitivity and specificity^(16)^.

Sensitivity is understood as the percentage value that corresponds to the alterations correctly predicted as positive, indicating the number of patients diagnosed with some alteration. Thus, it is calculated as resulting in true positives (true positives + false negatives). Specificity, in turn, quantifies the percentage of children who were correctly diagnosed without alterations in the absence of the condition^(17)^.

The positive predictive value (PPV) can be considered the percentage of individuals who are truly affected, whose alteration was identified by the test (true positives + false positives). The negative predictive value (NPV) can be defined as the percentage of healthy individuals in whom the test did not identify an alteration (true negatives + false positives)^(18)^.

For the analysis of results, the software R Core Team 2022 (Version 4.2.1) was used, and the data are presented objectively, where AUC indicates the area under the ROC curve. A 95% confidence interval (95% CI) was used. SE indicates sensitivity, ES specificity, followed by ACC, which represents accuracy, and the values of TP – True Positive, FN – False Negative, FP – False Positive, and TN – True Negative. The Constrained Maximum Sensitivity technique was applied, and the indicators of PPV and NPV were included.

Regarding the criteria for classifying the values found, due to the small number of data per group, the values and terminology of the results were adapted to the reality of the study. Thus, the values from the reference study^(17)^ classify 0.5–0.6 as “Unsatisfactory,” 0.6–0.7 as “Satisfactory,” 0.7–0.8 as “Good,” 0.8–0.9 as “Very Good,” and 0.9–1.0 as “Excellent.” In this study, however, the results were defined as follows: below 60% as “not ideal,” between 60% and 80% as “reasonable,” and above 80% as “ideal”^(19)^.

## RESULTS

The results were obtained through the analysis of two age groups: G1 (age range of 6 to 11 months and 29 days) and G2 (age range of 12 to 23 months and 29 days). G1 was composed of 40 cases and G2 of 36 cases.

Speech-language pathologists were asked to complete all reports related to the shared cases within 30 days. However, due to intercurrences such as delays and withdrawals of professionals, this period had to be extended to 150 days. During the course of the study, it was necessary to include new evaluators to complement or replace those who, for external or personal reasons, declined participation during the analyses. Of the 13 initial consents, only eight speech-language pathologists ultimately participated in the study, necessitating that some professionals contribute to the analysis of pending cases to complete the trio for each case analyzed. Analyses already completed by specialists who had not covered all cases in the group were not disregarded, provided that the given case had been fully analyzed by them. Thus, only cases that had not yet been analyzed in a given group were reassigned to another specialist already participating in the study. On average, each specialist analyzed between 31 and 40 cases (not all analyzed every case within a given group), including static and dynamic images of the OM evaluation, which comprised the study database.

During the study, the socio-professional profile of the clinicians linked to the “committee of specialist speech-language pathologists (evaluators)” of the gold standard test was outlined, with the data presented in Table 1.

**Table 1:** Socioprofessional profile of the committee of specialist speech-language pathologists

Age Group	% (n)
31 to 40 years	37.5 (3)
41 to 50 years	50.0 (4)
51 to 60 years	12.5 (1)
Region of professional practice	
North	12.5 (1)
Northeast	37.5 (3)
Middle-West	25.0 (2)
Southeast	25.0 (2)
Degree	
Postdoctoral	12.5 (1)
Doctorate	25.0 (2)
Master	37.5 (3)
Specialist	25.0 (2)
Teaching (time in practice)	
Less than 5 years	25 (2)
Between 5 and 10 years	37.5 (3)
Between 11 and 15 years	12.5 (1)
Between 21 and 25 years	12.5 (1)
Not Teaching	12.5 (1)
Years of experience in Speech-Language Pathology in orofacial motricity	
Between 05 and 10 years	37.5 (3)
Between 16 and 20 years	25.0 (2)
Between 21 and 25 years	25.0 (2)
More than 25 years	12.5 (1)
Years of practice in Orofacial Motricity with infants	
Less than 5 years	12.5 (1)
Between 5 and 10 years	25.0 (2)
Between 11 and 15 years	12.5 (1)
Between 16 and 20 years	25.0 (2)
Between 21 and 25 years	12.5 (1)
More than 25 years	12.5 (1)

**Caption:** % = Percent; n = Nº of observations

**Figure 1:**
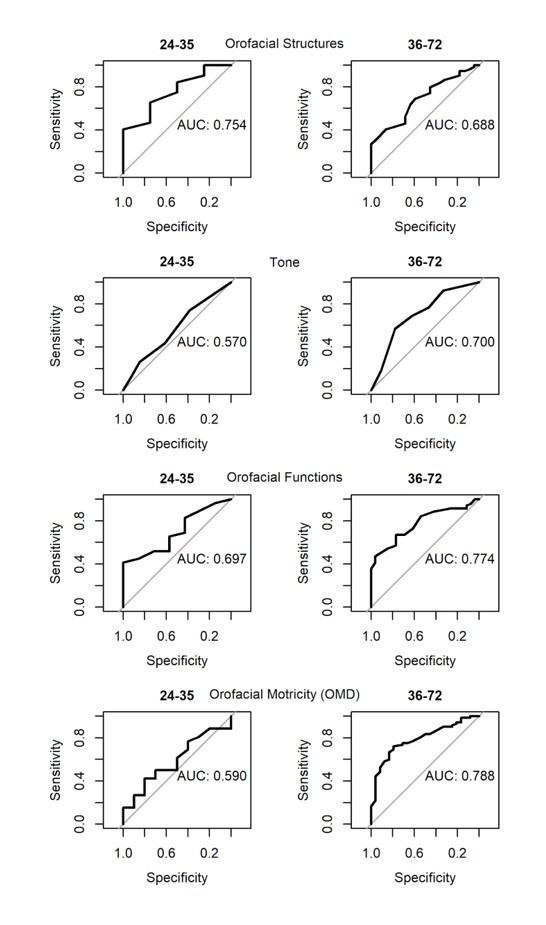
Graphical representation of the ROC curves for the domains of orofacial myofunctional assessment in infants and referrals, according to age group (in months)

For statistical purposes, domains were established for the analysis of OM in infants by age group, namely: orofacial structures (extraoral and intraoral), tone, orofacial functions (breathing, sucking, swallowing and/or chewing); as well as domains concerning alterations that indicate OM disorders and the need for referral for speech-language therapy due to OM disorders and/or other multidisciplinary referrals. Regarding the domains related to orofacial myofunctional evaluation, Table 2 presents the values obtained from the analysis of the reports issued by the committee of specialists, with the respective graphical representation of the ROC curve in Figure 1.

**Table 2:** Presentation of diagnostic accuracy values, according to sensitivity and specificity values, for the domains of orofacial myofunctional assessment in infants

Domain	Age Group (months)	AUC (95% IC)	Cutoff	Maximum Score	SE	SP	ACC	TP	FN	FP	TN	PPV	NPV
Orofacial Structures	6-11	0.609 (0.396-0.822)	7	62	83.3	28.6	45.0	10	2	20	8	33.3	80
	12-23	0.587 (0.360-0.815)	8	62	78.6	37.5	69.4	22	6	5	3	81.5	33.3
Tone	6-11	0.634 (0.454-0.815)	1	6	66.7	71.0	70	6	3	9	22	40	88
	12-23	0.672 (0.467-0.877)	1	6	71.4	50	66.7	20	8	4	4	83.3	33.3
Orofacial Functions	6-11	0.645 (0.369-0.920)	5	46	83.3	35.3	42.5	5	1	22	12	18.5	92.3
	12-23	0.586 (0.368-0.805)	5	92	96.3	0	72.2	26	1	9	0	74.3	0.0
Orofacial Motricity (OM disorders)	6-11	0.496 (0.298-0.694)	9	114	81.2	8.3	37.5	13	3	22	2	37.0	40.0
	12-23	0.602 (0.317-0.886)	14	160	96.9	25	88.9	31	1	3	1	91.2	50.0

**Caption:** AUC = Area under the ROC curve; 95% CI = 95% Confidence Interval; SE = Sensitivity; SP = Specificity; ACC = Accuracy; TP = True Positive; FN = False Negative; FP = False Positive; TN = True Negative. Constrained Maximum Sensitivity; PPV = Positive Predictive Value; NPV = Negative Predictive Value; OM disorders = Orofacial Myofunctional Disorders

A summary of the Orofacial Myofunctional Evaluation Protocol MMBGR – Infants and Preschoolers used to record orofacial myofunctional assessment in infants is presented in Table 2, with the inclusion of the respective cutoff points obtained in the present study (Chart 2).

**Chart 2:** Summary of the Orofacial Myofunctional Examination – MMBGR – Infants and Preschoolers, with inclusion of cutoff values by domains in infants, according to age group

**Age (months)**	**6-11**	**12-23**
EXTRAORAL EXAM (best result = 0 and worst = 20)		
Face *(best result = 0 and worst = 10)*	[ ]	[ ]
Lips (*best result = 0 and worst = 9)*	[ ]	[ ]
Mandible (*best result = 0 and worst = 1)*	[ ]	[ ]
INTRAORAL EXAM (best result = 0 and worst = 42/56)		
Lips *(best result = 0 and worst = 5)*	[ ]	[ ]
Cheeks *(best result = 0 and worst = 6/8)*	[ ]	[ ]
Tongue *(best result = 0 and worst = 13/16)*	[ ]	[ ]
Palate *(best result = 0 and worst = 10)*	[ ]	[ ]
Palatine tonsils *(best result = 0 and worst = 4)*	[ ]	[ ]
Teeth and occlusion *(best result = 0 and worst = 4/13)*	[ ]	[ ]
**RESULT OF EXTRAORAL + INTRAORAL EXAMINATION**	**[ ]**	**[ ]**
CUTOFF POINT: EXTRAORAL + INTRAORAL EXAMINATION	7	8
TONE (best result = 0 and worst = 6)		
Lips *(upper+lower) (best result = 0 and worst = 2)*	[ ]	[ ]
Mental *(best result = 0 and worst = 1)*	[ ]	[ ]
Tongue *(best result = 0 and worst = 1)*	[ ]	[ ]
Cheeks *(right+left) (best result = 0 and worst = 2)*	[ ]	[ ]
**RESULT OF TONE**	**[ ]**	**[ ]**
CUTOFF POINT: TONE	1	1
OROFACIAL FUNCTIONS (best result = 0 and worst = 46/92/53/68)		
Breathing *(best result = 0 and worst = 2)*	[ ]	[ ]
Suction/Swallowing *(best result = 0 and worst = 22)*	[ ]	[ ]
Chewing *(best result = 0 and worst = 13)*	___	[ ]
Swallowing semi-solid/solid *(best result = 0 and worst = 17)*	___	[ ]
Swallowing pasty *(best result = 0 and worst = 22)*	[ ]	[ ]
Swallowing liquid *(best result = 0 and worst = 15)*	___	[ ]
**RESULTS OF OROFACIAL FUNCTIONS**	**[ ]**	**[ ]**
CUTOFF POINT: OROFACIAL FUNCTIONS	5	5
**TOTAL SCORE (Orofacial Myofunctional Clinical Examination)**	**[ ]**	**[ ]**
CUTOFF POINT: Orofacial Myofunctional Disorder (OMD)	9	14

Regarding the domains related to referrals, Table 3 presents the values obtained from the analysis of the reports issued by the committee of specialists.

**Table 3:** Presentation of diagnostic accuracy values, according to sensitivity and specificity values, referring to the domains of referrals for infants

Domain	Age Group (months)	SE	SP	ACC	TP	FN	FP	TN	PPV	NPV
Referral to Speech-Language Pathology	6-11	13.3	84.0	57.5	2	13	4	21	33.3	61.8
	12-23	18.2	100	50.0	4	18	0	14	100	43.7
Other multidisciplinary referrals	6-11	44.4	87.1	77.5	4	5	4	27	50	84.4
	12-23	50	78.6	61.1	11	11	3	11	78.6	50

**Caption:** AUC = Area under the ROC curve; SE = Sensitivity; SP = Specificity; ACC = Accuracy; TP = True Positive; FN = False Negative; FP = False Positive; TN = True Negative. Constrained Maximum Sensitivity; PPV = Positive Predictive Value; NPV = Negative Predictive Value; OM disorders = Orofacial Myofunctional Disorders

To summarize the findings presented in the results and discussion, Table 4 shows the relationship between the diagnostic accuracy value and the classification for each infant age group of the MMBGR – Infants and Preschoolers protocol.

**Table 4:** Summary of diagnostic accuracy values (%) and classification, according to the domains of orofacial myofunctional assessment of infants and referrals, by age group

Domain	%Accuracy/Concept 06 to 11 months	%Accuracy/Concept 12 to 23 months
Orofacial Structures	45.0 - Not ideal	69.4 - Reasonable
Tone	70.0 – Reasonable	66.7 - Reasonable
Orofacial Functions	42.5 - Not ideal	72.2 - Reasonable
Orofacial Motricity (OMD)	37.5 - Not ideal	88.9 – Ideal
Referral to Speech-Language Pathology	57.5 - Not ideal	50.0 - Not ideal
Other Referrals (Multidisciplinary)	77.5 – Reasonable	61.1 - Reasonable

**Caption:** OMD = Orofacial Myofunctional Disorders

## DISCUSSION

The Orofacial Myofunctional Evaluation Protocol MMBGR – Infants and Preschoolers is the first assessment instrument in the field of OM to cover the age range from six to 71 months, allowing the assignment of scores for the purpose of diagnosing individuals with OM disorders. According to the specificities involved, it was considered appropriate to present the data exclusively for the infant population, including a breakdown by subgroups according to age range (Group 1: six to 11 months and 29 days; Group 2: 12 to 23 months and 29 days).

The academic degrees and experience of the evaluators who composed the committee of specialists ranged from specialization in OM to postdoctoral training, with extensive expertise in clinical care for infants in the field of OM (the majority, 87.5%, with more than five years of practice, and half with more than 16 years). This indicates that the images were analyzed by highly qualified professionals with a differentiated perspective for this population. The diversity of the committee of specialists covered four regions of the country, and in the formation of each trio for participant analysis, the evaluators came from different states. This allowed the instrument’s analysis to encompass perspectives and experiences from various regions of Brazil. The study's limitation in reaching the South region stemmed from the withdrawal of some speech-language pathologists, as previously mentioned in the results.

It is considered that the profile of the committee of specialists, which encompassed extensive teaching and clinical experience in OM with infants, enabled the image analyses to be carried out with the rigor required by the procedures designed for validation. This enhances the clinical value of the instrument’s content in assessing patients effectively and contributing to the scientific rigor of the study^(4)^.

With regard to the domain of orofacial structures in the Orofacial Myofunctional Evaluation Protocol MMBGR – Infants and Preschoolers, the results obtained for infants were classified as ideal (83.3%) and reasonable (78.6%) for sensitivity, indicating that the instrument is able to identify subjects with alterations in these structures when present. Specificity, however, was considered not ideal (28.6% and 37.5%), which indicates that the test is not very specific in predicting infants without alterations in their absence.

The diagnostic accuracy for the domain of orofacial structures was considered not ideal for the first age group and reasonable for infants over 12 months, indicating that the instrument is sufficiently capable of detecting predicted subjects with alterations in older infants. In the 6- to 11-month age group, however, the proportion of NPV was more evident than in the 12- to 23-month age group. This indicates that for the first age group, the instrument is more effective in identifying infants without alterations.

In fact, the need for adjustments in the position and posture of infants during image acquisition may have been a limiting factor and, therefore, may have influenced the instrument’s ability to identify infants predicted with alterations. Real-time assessment recording should be considered for the younger age group^(4)^. On the other hand, consideration is given to the possibility of expanding the indication of complementary examinations, such as ultrasonography, to support orofacial myofunctional assessment and diagnosis in infants.

As for the results indicating that the domain of orofacial structures is more accurate in the evaluation of individuals over 12 months than in the six to 11 months age group, it is considered that making some adjustments in the infants’ position and posture during image acquisition may be a useful resource, in addition to recording the protocol in face-to-face situations.

For the domain of tone, sensitivity levels were above 60% for both age groups, being considered reasonable, indicating that the instrument is sufficient for detecting infants with alterations. Specificity was reasonable for the first age group (71%) but not ideal for those over 12 months, indicating that the first group showed more sufficient values in identifying subjects without alterations in their absence. It is considered that the evaluation obtained through video analysis in the present study may have hindered the diagnosis of tone alterations, since the evaluator is deprived of proprioceptive information. The method of video image analysis is limited in this domain; therefore, it is recommended that, in clinical practice, the authors’ guidance be followed to use palpation at the time of assessment^(5,6)^. Real-time recording of tone could minimize possible difficulties in analysis. Nevertheless, diagnostic accuracy in the tone domain remained reasonable for all age groups; therefore, the instrument showed values considered sufficient in accurately identifying what it was intended to assess regarding tone.

Orofacial functions showed sensitivities considered ideal (83.3% and 96.3%) for all infant age groups; therefore, the instrument is effective in identifying subjects with alterations when present. Specificity, however, was considered not ideal for orofacial functions, as it was less effective in identifying subjects without alterations in their absence. In the six to 11 months age group, subjects predicted without alterations in orofacial functions were better identified (NPV of 92.3%) than those predicted with alterations.

Diagnostic accuracy in the domain of orofacial functions remained reasonable only for older infants. This could be explained by the physiological maturation process of the speech articulatory organs from birth, in which alterations and “atypical” patterns become more evident with increasing age. The differences are less subtle as the musculature becomes more structured and developed; therefore, adaptations that might have been tolerated in younger infants should already give way to standard execution^(20)^.

The domain referring to OM alteration concerns the presence or absence of OM disorders in infants. Ideal sensitivity values were obtained (81.2% and 96.9%) and specificity was not ideal for all infant ages; therefore, the instrument proved sufficient to identify subjects with alterations but insufficient to rule out those without alterations.

The diagnostic accuracy results for OM disorders were not ideal in infants aged 6 to 11 months but ideal in those aged 12 to 23 months, with higher PPV values (91.2%) in the latter age group. The findings indicate that, even with ideal sensitivity, the first group had a lower ability to predict subjects with alterations, which may be explained by the fact that atypical developmental processes can be more subtle at earlier ages, whereas at later ages, due to changes in physical, neurological, cognitive, and behavioral structures, these processes may become more evident^(21)^.

Regarding the cutoff score values obtained for each specific domain of the MMBGR – Infants and Preschoolers protocol, it is considered that even low values are already indicative of alterations in the domains evaluated^(22)^. For example, regarding orofacial structures, the instrument allows scores ranging from 0 (best result) to 62 (worst result), with cutoff scores of 7 for G1 and 8 for G2. The same applies to tone, which ranges from 0 to 6, where obtaining 1 point already indicates an alteration, and to orofacial functions, which range from 0 to 46 for G1 and from 0 to 92 for G2, with a cutoff score of 5 as an indicator of alteration. Thus, low scores in the specific domains should already require special attention from the clinician regarding aspects involving orofacial myofunctional alterations in infants.

In the same way as in the specific domains, regarding the total scores for OM disorders, the values of 9 (infants aged six to 11 months and 29 days) and 14 (infants aged 12 to 23 months and 29 days) already point to the importance of the clinician’s attention to OM disorders and the corresponding need for speech-language and/or multidisciplinary follow-up. The evaluation can provide support for rehabilitation and referrals in the early stages of life, which are crucial for the growth and development of the stomatognathic system^(23)^. The lack of timely speech-language assessment and/or intervention may lead to consequences that persist into adulthood^(9)^.

Alterations identified in the first year of life have a greater likelihood of resolution^(24)^. In addition, given the relationship of Speech-Language Pathology with other health fields, professionals must be able to indicate multidisciplinary follow-up^(10)^. However, although the needs (or not) for speech-language follow-up and/or referrals to other professionals were treated as domains for calculating diagnostic accuracy, the central point of the protocol is that aspects inherent to oromyofunctional assessment be recorded, which include structures (static and mobile), tone, and the respective orofacial functions with score assignments.

The need for referral to Speech-Language Pathology was evaluated as an important domain for the rehabilitation of individuals predicted to have alterations. In both infant groups, specificity (84.0% and 100%) was higher than sensitivity, indicating that individuals without the need for referral were better identified than those requiring it, especially in the 6- to 11-month age group (PPV of 61.8%). For both infant groups, the diagnostic accuracy regarding the indication of speech-language follow-up was classified as not ideal. It is considered that at older ages, possible orofacial myofunctional adaptations are less tolerated by the clinician, resulting in a greater occurrence of referrals. Furthermore, as in other domains previously discussed, this finding may be related to the validation study design, in which analyses were performed from recorded image data rather than real-time assessment situations.

The last domain evaluated, concerning the need for other multidisciplinary referrals (E.g., Dentistry, Otorhinolaryngology), showed sensitivity and specificity values similar to those related to referral to Speech-Language Pathology. Although both infant groups had non-ideal sensitivity values, in the six-to-11-month age group, the NPV was ideal (84.4%), which is considered important to avoid unnecessary referrals at a stage of life that already requires many general and specific childcare measures. In infants over 12 months, the PPV was considered reasonable (78.6%) for predicting the need for multidisciplinary referral, which is fundamental for guiding treatment, as the absence of such referral may represent a risk situation in the presence of an undiagnosed and untreated OM disorder^(25)^. It is emphasized that diagnostic accuracy remained reasonable for all age groups, mainly due to the nature of the items analyzed, with a predominance of static images (Dentistry: teeth and occlusion; Otorhinolaryngology: lingual frenulum, palatine tonsils, and marking of nasal flow using the mirror).

Despite the difficulties in recruiting a committee of specialist speech-language pathologists, all activities were fully completed to achieve the objective of determining the cutoff point for OM disorders. The main limitation of the study refers to the nature of the data analysis methodology in validation studies, which involves analyzing cases based on recorded images (both static and dynamic). This approach is not always ideal for evaluating certain domains. It is considered that, when using the MMBGR – Infants and Preschoolers protocol in clinical speech-language practice, the speech-language pathologist may apply the protocol with real-time recording, in addition to later reviewing the images obtained from the infant, which may possibly contribute to achieving ideal accuracy in the items/domains that compose the diagnosis of OM disorders.

The limitations of diagnostic instruments for OM disorders could be reduced with the expansion of protocols capable of assessing OM in the early stages of life, noting that the number of instruments specifically designed for this age group remains limited. As a future perspective, it is considered that the use of other observable parameters to compose orofacial myofunctional assessments in infants, including the use of instrumental examinations such as ultrasonography, may be valuable^(26,27)^ and may become an interesting alternative to complement the diagnosis of OM disorders. It should be emphasized, however, that the identification of individuals with alterations remains the main objective of the MMBGR – Infants and Preschoolers protocol.

## CONCLUSION

The Orofacial Myofunctional Evaluation Protocol MMBGR – Infants and Preschoolers is a resource for use in clinical and research settings in Speech-Language Pathology, for establishing diagnoses in the early stages of life. The definition of cutoff points allows the speech-language pathologist to recognize OM disorders, use them as parameters for reassessments, and contribute to the longitudinal follow-up of patients.

The Orofacial Myofunctional Evaluation Protocol MMBGR – Infants and Preschoolers, when analyzed through photos and videos, is accurate for infants aged six to 11 months only in the domain of tone, and for infants aged 12 to 23 months in all examination domains, being ideal for diagnosing OM disorders. The instrument is reasonable regarding the need for multidisciplinary referrals for the entire infant age range.

The study on cutoff points in the MMBGR – Infants and Preschoolers protocol is a milestone for clinical and research practice in Speech-Language Pathology, as it is an important instrument in Brazil in the field of OM. Furthermore, the present research may serve as a guide for new studies involving orofacial myofunctional assessment in the early stages of life.
