# Supplementary material for: MMBGR protocol – diagnostic accuracy of clinical examination in infants
Source: Codas. 2025 Dec 8;37(6):e20240315. doi: 10.1590/2317-1782/e20240315en (PMC12704608; doi:10.1590/2317-1782/e20240315en)
Supplement: Translation

# Translation: Protocolo MMBGR - acurácia diagnóstica do exame clínico em lactentes

**DOI:** 10.1590/2317-1782/e20240315pt

**Authors:** Anna Luiza dos Santos Matos, Giédre Berretin-Felix, Íkaro Daniel de Carvalho Barreto, Ricardo Queiroz Gurgel, Andréa Monteiro Correia Medeiros

**Affiliations:** 1 Programa de Pós-graduação em Ciências da Saúde, Universidade Federal de Sergipe – UFS - Aracaju (SE), Brasil.; 2 Departamento de Fonoaudiologia, Faculdade de Odontologia de Bauru, Universidade de São Paulo – USP - São Paulo (SP), Brasil.; 3 Centro Brasileiro de Pesquisa em Avaliação e Seleção e de Promoção de Eventos – CEBRASPE - Brasília (DF), Brasil.; 4 Departamento de Fonoaudiologia, Universidade Federal de Sergipe – UFS - São Cristóvão (SE), Brasil.

**Keywords:** Fonoaudiologia, Lactente, Diagnóstico Clínico, Estudos de Validação, Sensibilidade e Especificidade, Terapia Miofuncional, Sistema Estomatognático

## Abstract

**Objetivo:**

Apresentar acurácia diagnóstica do Protocolo de Avaliação Miofuncional Orofacial MMBGR Lactentes e Pré-escolares, faixa etária de seis a 23 meses.

**Método:**

Validação da acurácia diagnóstica, com amostra por conveniência. Analisadas imagens de 76 participantes, por trio de fonoaudiólogos especialistas, de modo individual e separadamente, sendo considerada válida concordância entre dois deles. Utilizado formulário eletrônico quanto aos domínios do exame miofuncional orofacial, Distúrbio Miofuncional Orofacial (DMO) e necessidade de encaminhamentos. Foram comparadas as respostas dos fonoaudiólogos que emitiram pareceres baseados na experiência clínica, sem utilizar o protocolo (padrão ouro) com àquelas emitidas por fonoaudiólogos utilizando o Protocolo (teste índice). Utilizado método Receiver Operating Characteristic Curve (ROC), atribuídos pontos de corte e obtidos valores de sensibilidade e especificidade; software R Core Team 2022.

**Resultados:**

Para lactentes de seis a 11 meses a acurácia diagnóstica é não ideal para estruturas e funções orofaciais e DMO, e razoável para tônus; em lactentes entre 12 e 23 meses, acurácia é razoável para estruturas e funções orofaciais, tônus, e ideal para DMO. O ponto de corte para DMO é 9 (seis a 11 meses) e 14 (12 a 23 meses). A acurácia é razoável quanto aos encaminhamentos multidisciplinares.

**Conclusão:**

Protocolo de Avaliação Miofuncional Orofacial MMBGR – Lactentes e Pré-escolares, quando analisado por meio de fotos e vídeos, é acurado para lactentes entre seis a 11 meses somente quanto ao tônus; e para lactentes de 12 a 23 meses em todos os domínios do exame, sendo ideal para diagnosticar DMO. Mostrou-se razoável quanto à necessidade de encaminhamentos multidisciplinares.

## INTRODUÇÃO

A expansão da Fonoaudiologia gerou a necessidade de avanços científicos e tecnológicos, o que inclui a área de Motricidade Orofacial (MO), que é responsável pela promoção, prevenção, diagnóstico e reabilitação de estruturas orofaciais e suas funções, especificamente respiração, sucção, mastigação, deglutição e fala^(1)^. Associado ao crescimento da área, a necessidade de diagnósticos em tempo oportuno ganhou força, principalmente com o olhar voltado ao público infantil, visto que a avaliação antecipada pode fornecer subsídios para reabilitação e encaminhamentos em idades iniciais da vida, bem como a estruturação de serviços, apoio e recursos, evitando maiores dificuldades em idades posteriores^(2)^.

Com intenção de padronizar a avaliação fonoaudiológica, especialmente nos casos que exigem intervenções ainda na infância, o Conselho Federal de Fonoaudiologia (CFFa), por meio da resolução de número 414, determinou que seja preconizado o uso de instrumentos na prática clínica para diagnóstico e tratamento de transtornos da comunicação, estes são protocolos, testes, softwares e outros recursos^(3)^. Atualmente já existem alguns protocolos reconhecidos para o faixa etária de lactentes (um a 23 meses de vida) com algumas fases de validação realizadas, como por exemplo, os protocolos AMIOFE-E Lactentes^(4)^ e o MMBGR - Lactentes e Pré-escolares^(5-7)^.

Um instrumento validado permite o registro de dados de modo confiável e preciso, possibilitando a análise detalhada dos resultados, que pode contribuir no atendimento ao paciente, na discussão de casos entre os diversos profissionais da saúde, com intenção de servir como documento de análise padronizada do indivíduo^(7,8)^. Apesar da suma importância, o número de protocolos avaliativos na clínica da MO, voltado para pediatria, ainda é restrito, o que reforça a necessidade de ampliação das pesquisas para criação e validação de instrumentos voltados para idades iniciais.

A falta de avaliação e/ou intervenção fonoaudiológica pode implicar em consequências até a vida adulta, tais como dificuldades respiratórias, alimentares e comunicativas^(9)^. Além disso, dada a relação da Fonoaudiologia com as demais áreas da saúde, como Medicina, Odontologia, Fisioterapia, Terapia Ocupacional e Psicologia, os profissionais devem ser capazes de contribuir para uma avaliação sistematizada, rigorosa e completa, principalmente em casos de acompanhamentos multidisciplinares^(10)^.

Destaca-se a potencialidade do Protocolo MMBGR-Lactentes e Pré-escolares em registrar características tidas como alteração, em especial miofuncionais orofaciais, de modo a padronizar a avaliação, guiar o planejamento terapêutico e servir como documentação para a atuação e encaminhamentos multidisciplinares^(5,6)^. Este foi publicado apresentando as etapas de validação de conteúdo, processos de respostas e confiabilidade, porém até o presente momento não havia sido realizada a etapa de validade de acurácia diagnóstica, com medidas definidoras do diagnóstico^(11)^ e que também colaborem na decisão de encaminhamentos.

A acurácia diagnóstica mensura como determinado teste é capaz de identificar corretamente os indivíduos com alteração e excluir aqueles que não as têm. O resultado do teste pretendido (teste índice) deve ser comparado com o resultado de um teste considerado padrão de referência (padrão ouro). A comparação destes testes deve indicar a preditividade, referente à capacidade do teste prever resultados de modo antecipado, o que considera sensibilidade, especificidade e valores preditivos^(12,13)^.

O presente estudo apresenta a fase de validação da acurácia diagnóstica referente à faixa etária de lactentes do Protocolo MMBGR - Lactentes e Pré-escolares: Exame Clínico Miofuncional Orofacial com escores; e a necessidade de encaminhamentos fonoaudiológicos e/ou áreas afins.

## MÉTODO

Estudo do tipo validação referente à acurácia diagnóstica, prospectivo e por conveniência, com anuência das autoras do protocolo original. Foi aprovado pelo Comitê de Ética em Pesquisa (CEP) da Universidade Federal de Sergipe sob número de parecer 5.147.320 e abrange a descrição da etapa de validação da acurácia diagnóstica do Protocolo de Exame Clínico Miofuncional Orofacial – MMBGR - Lactentes e Pré-escolares^(5)^, referente à faixa etária de seis a 23 meses de idade.

Para compreender a etapa de validação da acurácia diagnóstica aqui apresentada, é necessário sinalizar que ela está relacionada às etapas de validação que foram realizadas em anos anteriores, em atividade de pós-doutoramento executada pela coordenadora/orientadora da pesquisa, ocasião que foram obtidos os termos de consentimentos das instituições e responsáveis pelos lactentes, mediante a assinatura prévia do termo de consentimento livre e esclarecido (TCLE).

Os fonoaudiólogos foram recrutados a partir da análise de seus currículos lattes, conforme os critérios de inclusão da pesquisa, cujo profissional deveria ser “especialista em Motricidade Orofacial”, a partir da busca de profissionais elencados como especialistas no site do CFFa, por diferentes regiões do país. A partir desse levantamento, foi feito contato com cada profissional, os quais foram convidados a participar do projeto por meio do contato prévio da pesquisadora responsável, por aplicativo de mensagens instantâneas e por meio eletrônico (e-mail). Aqueles que manifestaram interesse, receberam via e-mail os materiais contendo TCLE e *link* para impressão dele, conforme preconizado pelo CEP.

Ainda por e-mail, foi encaminhado o *link* para o formulário de caracterização profissional, onde a anuência para participação do estudo deveria ser sinalizada, e outro *link* correspondente ao preenchimento da avaliação referente aos casos clínicos a serem analisados. Foi realizado o compartilhamento da nuvem que continha imagens estáticas e dinâmicas dos lactentes a serem avaliados.

### Etapas anteriores

O Protocolo MMBGR - Lactentes e Pré-escolares já havia passado por duas etapas de validação, a saber: validade de conteúdo do instrumento e validade baseada nos processos de respostas com aplicação do exame clínico e realização da confiabilidade do teste^(5,6)^.

Na ocasião da aplicabilidade do exame clínico, foi construído um banco de dados com imagens estáticas e dinâmicas dos lactentes que frequentavam instituições (hospital universitário e creches) nos estados de São Paulo e Sergipe, que atendiam aos critérios de inclusão, que eram não possuir queixas fonoaudiológicas, diagnósticos neurológicos ou transtornos do desenvolvimento^(5)^.

### Constituição dos grupos de participantes (lactentes)

As imagens dos participantes foram distribuídas em dois grupos por faixas etárias: G1, grupo correspondente à faixa de seis a 11 meses e 29 dias de vida, com 40 casos; e G2 de 12 a 23 meses e 29 dias, com 36 casos.

A distribuição dos grupos por faixa etária seguiu a mesma padronização do protocolo original5, inclusive quanto aos itens e domínios a serem avaliados por idade. Assim, todos os itens analisados eram comuns às duas faixas etárias, exceto mastigação que no grupo de 12 a 23 meses foi feita a avaliação com a introdução de alimentos na consistência sólida, o que não ocorreu no grupo mais jovem. Os autores consideram que existe um processo gradual de modificação na aceitação da alimentação, sendo que entre os 12 e os 24 meses há a exploração de diferentes texturas e sabores^(14)^.

Quantos aos domínios citados, o exame extraoral continha itens relacionados à face, aos lábios e a mandíbula; o exame intraoral, direcionado à mucosa dos lábios, bochechas, língua, palato, tonsilas palatinas e dentes. Havia ainda a emissão de opinião acerca dos dados relacionados aos tônus de lábios, língua, região do mento e bochechas. Para análises das funções estomatognáticas, foram disponibilizadas as imagens dinâmicas das funções de respiração, sucção, deglutição de líquido, semissólido e sólido e mastigação. Além das análises que envolviam os domínios miofuncionais orofaciais, ao final do formulário, o avaliador deveria indicar no domínio geral se o participante avaliado apresentara ou não DMO, se houve dificuldade para preenchimento do formulário e se havia necessidade de encaminhamentos fonoaudiológicos e/ou multidisciplinares (Odontologia, Otorrinolaringologia, entre outros).

No Quadro 1 está descrito o que foi proposto para a ser analisado pelo fonoaudiólogo, respeitando o grupo etário que o lactente fazia parte.

**Quadro 1:** Itens avaliados pelos fonoaudiólogos, por faixa etária de lactentes

Faixa Etária	Itens Avaliados
06-11 meses	✔ Estruturas Orofaciais (intra e extraorais)
	✔ Tônus
	⮚ Respiração
	⮚ Sucção/Deglutição (mamadeira ou peito)
	⮚ Deglutição de pastoso (papa, purê ou alimento amassado)
12-23 meses	✔ Estruturas Orofaciais (intra e extraorais)
	✔ Tônus
	⮚ Respiração
	⮚ Sucção (mamadeira ou peito)
	⮚ Mastigação: sólido/semissólido
	⮚ Deglutição: pastoso (papa, purê, alimento amassado); sólido ou semissólido (alimento contendo pedaços cortados bem pequenos e macios ou desfiados); e líquido

### Acurácia Diagnóstica: teste índice e teste padrão ouro

#### Teste índice

Na etapa anterior referente à validade baseada nos processos de respostas, com a realização da confiabilidade do teste^(5,6)^, fonoaudiólogos calibrados avaliaram as imagens obtidas dos lactentes com o uso do Protocolo MMBGR - Lactentes e Pré-escolares, sendo verificada a concordância entre e intra avaliador^(5)^ de cada item do instrumento. Essas avaliações (armazenadas no banco de dados do pesquisador) foram consideradas no presente estudo como “teste índice”.

#### Teste Padrão Ouro

Fonoaudiólogos especialistas em MO com experiência clínica com lactentes e que não tivessem participado das etapas anteriores de validação do Protocolo MMBGR - Lactentes e Pré-escolares, compuseram a análise determinada como “teste padrão ouro”. No total foram oito especialistas, que ao aceitarem participar formaram o “comitê de fonoaudiólogos especialistas (avaliadores)” do grupo “padrão ouro”, os quais realizaram o registro de sua caracterização profissional e as análises das imagens, de modo individual e separadamente, sem o uso do Protocolo MMBGR - Lactentes e Pré-escolares.

Os avaliadores responderam a dois formulários eletrônicos. O primeiro de Caracterização Profissional continha questões sobre: faixa etária, região de atuação, titulação, docência, anos de experiência e atuação com lactentes. O segundo referia-se ao exame clínico miofuncional orofacial, direcionado para incluir as respostas de avaliação dos casos, de forma a considerar domínios específicos e geral. Foi proposto que cada integrante (lactente) dos grupos G1 e G2 contasse com a avaliação de um trio de especialistas.

Na avaliação miofuncional orofacial os fonoaudiólogos do grupo padrão ouro emitiram pareceres sobre as mesmas imagens dos lactentes (banco de dados) sem utilizar o Protocolo MMBGR - Lactentes e Pré-escolares, baseando-se na sua experiência na área de MO, mediante os questionamentos: Quanto ao domínio apresentado foram encontradas alterações? Sim ou não. No grupo padrão ouro não foram atribuídos escores na avaliação, sendo que o avaliador apenas afirmava se havia ou não alteração.

Seguem os itens questionados.

Domínios:

Exame extraoral (face, lábios e mandíbula);

Exame intraoral (lábios, bochechas, língua, palato, tonsilas palatinas, dentes e oclusão);

Tônus (lábios, região do mento, língua e bochechas);

Funções orofaciais – G1 (respiração, sucção e deglutição de pastoso);

Funções orofaciais – G2 (respiração, sucção, mastigação, deglutição de líquido, pastoso, sólido e semissólido).

Aspectos gerais:

Essa criança possui alteração de Motricidade Orofacial?

Essa criança necessita de tratamento fonoaudiológico?

Há necessidade de encaminhamentos? (otorrinolaringologia, odontologia, outros)

Sobre o preenchimento:

Você teve algum problema para preencher esse formulário?

As respostas do especialista, referente a cada caso clínico, foram inseridas nos formulários eletrônicos disponibilizados. Foram tabuladas e armazenadas em uma plataforma digital, analisadas intragrupo, e esperada a concordância quanto à presença ou à ausência de alteração de MO de pelo menos dois dos três especialistas que analisaram cada caso.

### Análises para validação da Acurácia Diagnóstica

A proposta foi comparar os resultados do “teste padrão ouro” ao do “teste índice”. Assim, pretendeu-se definir como o processo avaliativo com a utilização do Protocolo de Avaliação Miofuncional Orofacial MMBGR - Lactentes e Pré-escolares (Teste índice) foi capaz de ser efetivo no momento que é comparado à avaliação clínica sem o uso do Protocolo MMBGR - Lactentes e Pré-escolares (Teste padrão ouro) e se seria capaz de prevê-lo.

Para que fosse validada a análise de acurácia diagnóstica, foi necessário especificar a preditividade. Logo, utilizou-se o método da curva *Receiver Operating Characteristic Curve* (ROC)^(13)^, pois, por meio da mesma, pode se reconhecer a área abaixo da curva, além de ser possível determinar pontos de corte sensível para alterações dos itens avaliados no Protocolo de Avaliação Miofuncional Orofacial MMBGR - Lactentes e Pré-escolares.

Com a detecção de cada ponto de corte, também foram calculados valores de sensibilidade e especificidade. Vale ressaltar que é possível não existir relação entre alteração de MO e os escores do Protocolo de Avaliação Miofuncional Orofacial MMBGR - Lactentes e Pré-escolares, por domínio (AUC=0,5) por meio do teste Z para proporções^(15)^. Sobre a análise, no espaço abaixo da curva (AUC) torna-se maior a probabilidade de identificação dos indivíduos que são verdadeiros positivos para DMO e os indivíduos que não são. Um teste ótimo terá uma AUC com valor próximo a 1,0. Portanto, quanto mais próximo de 1,0, maiores serão a sensibilidade e a especificidade^(16)^.

A sensibilidade é compreendida como o valor em porcentagem que corresponde às alterações que foram corretamente preditas como positivas, ou seja, indica o número de pacientes diagnosticados com alguma alteração. Dessa forma, é calculado como resultando em verdadeiros positivos (verdadeiros positivos + falsos negativos). Por sua vez, a especificidade quantifica em porcentagem o número de crianças que foram corretamente diagnosticadas sem alterações na ausência da alteração^(17)^.

O valor preditivo positivo (VPP) pode ser considerado como o percentual de indivíduos realmente alterado, cujo teste identificou alteração (verdadeiros positivos + falsos positivos). O Valor preditivo negativo (VPN) pode ser definido como a porcentagem de indivíduos sadios que o teste não identificou alteração (verdadeiros negativos + falsos positivos)^(18)^.

Para análise dos resultados foi utilizado o software R Core Team 2022 (Versão 4.2.1), e os dados seguem apresentados de forma objetiva, onde AUC indica Área abaixo da curva ROC. Utilizou-se IC95% – Intervalo com 95% de confiança. SE – sensibilidade, ES para especificidade, seguido de ACC, que representa Acurácia, e os valores de VP – Verdadeiro Positivo e FN – Falso Negativo, FP – Falso Positivo, VN – Verdadeiro Negativo. A técnica de Maximização da Sensibilidade com Especificidade mínima (*Constrained Maximum Sensitivity*) foi utilizada, e incluíram-se os indicativos de VPP e VPN.

A respeito dos critérios para classificação dos valores encontrados, devido ao baixo número de dados por grupo, foram adaptados valores e denominação dos resultados para realidade do estudo. Assim, os valores do Estudo de referência^(17)^ classifica: 0.5 - 0.6 em “Não Satisfatório”, 0.6 - 0.7 “Satisfatório”, 0.7 - 0.8 “Bom”, 0.8 - 0.9 “Muito Bom”, 0.9 - 1.0 ”Excelente”. Por sua vez, neste estudo atribuiu-se os resultados tal como: abaixo de 60% como “não ideal”, entre 60% e 80% como “razoável” e acima de 80% como “ideal”^(19)^.

## RESULTADOS

Os resultados foram obtidos conforme análise de dois grupos etários, chamados de G1 (faixa etária de seis a 11 meses e 29 dias) e G2 (faixa etária de 12 até 23 meses e 29 dias). G1 composto por 40 casos e G2 por 36 casos.

Foi solicitado que os fonoaudiólogos finalizassem todos os pareceres relacionados aos casos compartilhados no prazo de 30 dias. No entanto, devido a intercorrências, tais como atrasos e desistências dos profissionais, esse prazo precisou ser estendido para 150 dias. Ao decorrer do estudo, houve a necessidade de ingresso de novos avaliadores com função de complementar ou substituir aqueles que, por motivos extrínsecos/particulares, declinaram a participação no decorrer das análises. Do total de 13 anuências iniciais, participaram do estudo apenas oito fonoaudiólogos, sendo necessário que alguns profissionais colaborassem com as análises de casos pendentes, para compor o trio de cada caso analisado. As análises já concluídas pelos especialistas que não contemplaram todos os casos do grupo não foram desconsideradas, desde que determinado caso tivesse sido completamente analisado por este. Assim, somente os casos que não tinham sido ainda analisados em determinado grupo, foram passados para outro especialista já integrante da pesquisa. Em média, cada especialista analisou de 31 a 40 casos (nem todos analisaram todos os casos contidos em determinado grupo), incluindo imagens estáticas e dinâmicas da avaliação da MO, que compunham o banco de dados da pesquisa.

Durante o estudo, foi traçado o perfil socioprofissional dos clínicos que estavam vinculados ao “comitê de fonoaudiólogos especialistas (avaliadores)” do teste padrão ouro, cujos os dados seguem apresentados na Tabela 1.

**Tabela 1:** Perfil socioprofissional do comitê de fonoaudiólogos especialistas

Faixa etária	% (n)
31 e 40 anos	37,5 (3)
41 e 50 anos	50,0 (4)
51 e 60 anos	12,5 (1)
Região de exercício profissional	
Norte	12,5 (1)
Nordeste	37,5 (3)
Centro – oeste	25,0 (2)
Sudeste	25,0 (2)
Titulação	
Pós – Doutor	12,5 (1)
Doutor	25,0 (2)
Mestre	37,5 (3)
Especialista	25,0 (2)
Docência (tempo de exercício)	
Menos de 05 anos	25 (2)
Entre 05 e 10 anos	37,5 (3)
Entre 11 e 15 anos	12,5 (1)
Entre 21 e 25 anos	12,5 (1)
Não docente	12,5 (1)
Tempo de experiência em Fonoaudiologia na motricidade orofacial	
Entre 05 e 10 anos	37,5 (3)
Entre 16 e 20 anos	25,0 (2)
Entre 21 e 25 anos	25,0 (2)
Mais de 25 anos	12,5 (1)
Tempo de atuação na área de Motricidade Orofacial com lactentes	
Menos de 05 anos	12,5 (1)
Entre 05 e 10 anos	25,0 (2)
Entre 11 e 15 anos	12,5 (1)
Entre 16 e 20 anos	25,0 (2)
Entre 21 e 25 anos	12,5 (1)
Mais de 25 anos	12,5 (1)

**Legenda:** % = Percentual; n = número de observações

Por questões estatísticas, foram estabelecidos domínios para análise da MO dos lactentes por faixa etária, são estes: estruturas orofaciais (extra oral e intra oral), tônus, funções orofaciais (respiração, sucção, deglutição e/ou mastigação); além dos domínios quanto a alterações que indicam DMO e necessidade de encaminhamento para tratamento fonoaudiológico devido ao DMO e/ou outros encaminhamentos multidisciplinares. Quanto aos domínios pertencentes à avaliação miofuncional orofacial, segue na Tabela 2 os valores encontrados a partir da análise dos pareceres emitidos pelo comitê de especialistas, com respectiva representação gráfica da curva ROC na Figura 1.

**Tabela 2:** Apresentação dos valores de acurácia diagnóstica, conforme valores de sensibilidade e especificidade, referente aos domínios da avaliação miofuncional orofacial em lactentes

Domínio	Faixa Etária (meses)	AUC (IC95%)	Valor de Corte	Nota máxima	SE	ES	ACC	VP	FN	FP	VN	VPP	VPN
Estruturas orofaciais	6-11	0,609 (0,396-0,822)	7	62	83,3	28,6	45,0	10	2	20	8	33,3	80
	12-23	0,587 (0,360-0,815)	8	62	78,6	37,5	69,4	22	6	5	3	81,5	33,3
Tônus	6-11	0,634 (0,454-0,815)	1	6	66,7	71,0	70	6	3	9	22	40	88
	12-23	0,672 (0,467-0,877)	1	6	71,4	50	66,7	20	8	4	4	83,3	33,3
Funções Orofaciais	6-11	0,645 (0,369-0,920)	5	46	83,3	35,3	42,5	5	1	22	12	18,5	92,3
	12-23	0,586 (0,368-0,805)	5	92	96,3	0	72,2	26	1	9	0	74,3	0,0
Motricidade Orofacial (DMO)	6-11	0,496 (0,298-0,694)	9	114	81,2	8,3	37,5	13	3	22	2	37,0	40,0
	12-23	0,602 (0,317-0,886)	14	160	96,9	25	88,9	31	1	3	1	91,2	50,0

**Legenda:** AUC = Área abaixo da curva ROC; IC95% = Intervalo com 95% de Confiança; SE = Sensibilidade; ES = Especificidade; ACC = Acurácia; VP = Verdadeiro Positivo; FN = Falso Negativo; FP = Falso Positivo; VN = Verdadeiro Negativo; Maximização da Sensibilidade com Especificidade mínima (Constrained Maximum Sensitivity); VPP = Valor Preditivo Positivo; VPN = Valor Preditivo Negativo; DMO = Distúrbio Miofuncional Orofacial

**Figura 1:** Representação gráfica das curvas ROC dos domínios da avaliação miofuncional orofacial em lactentes e encaminhamentos, conforme faixa etária (em meses)

Segue o resumo do Exame do Protocolo de Avaliação Miofuncional MMBGR -Lactentes e Pré-escolares utilizado para registro da avaliação miofuncional orofacial em lactentes, com inserção dos respectivos pontos de corte obtidos no presente estudo. (Quadro 2)

**Quadro 2:** Resumo do Exame Miofuncional Orofacial - MMBGR - Lactentes e Pré-escolares, com inserção dos valores de ponto de corte por domínios em lactentes, conforme faixa etária

**Faixa etária (meses)**	**6-11**	**12-23**
EXAME EXTRAORAL (melhor resultado = 0 e pior = 20)		
Face (*melhor resultado = 0 e pior = 10)*	[ ]	[ ]
Lábios (*melhor resultado = 0 e pior = 9)*	[ ]	[ ]
Mandíbula (*melhor resultado = 0 e pior =1)*	[ ]	[ ]
EXAME INTRAORAL (melhor resultado = 0 e pior = 42)		
Lábios *(melhor resultado = 0 e pior = 5)*	[ ]	[ ]
Bochechas *(melhor resultado = 0 e pior = 6/8)*	[ ]	[ ]
Língua *(melhor resultado = 0 e pior = 13/16)*	[ ]	[ ]
Palato *(melhor resultado = 0 e pior = 10)*	[ ]	[ ]
Tonsilas palatinas *(melhor resultado = 0 e pior = 4)*	[ ]	[ ]
Dentes e Oclusão *(melhor resultado = 0 e pior = 4/13)*	[ ]	[ ]
**RESULTADO EXAME EXTRAORAL + INTRAORAL**	**[ ]**	**[ ]**
PONTO DE CORTE: EXAME EXTRAORAL + INTRAORAL	7	8
TÔNUS (melhor resultado = 0 e pior = 6)		
Lábios *(superior + inferior) (melhor resultado = 0 e pior = 2)*	[ ]	[ ]
Mento *(melhor resultado = 0 e pior = 1)*	[ ]	[ ]
Língua *(melhor resultado = 0 e pior = 1)*	[ ]	[ ]
Bochechas *(direita + esquerda) (melhor resultado = 0 e pior = 2)*	[ ]	[ ]
**RESULTADO TÔNUS**	**[ ]**	**[ ]**
PONTO DE CORTE: TÔNUS	1	1
FUNÇÕES OROFACIAIS (melhor resultado = 0 e pior = 46/92)		
Respiração *(melhor resultado = 0 e pior = 2)*	[ ]	[ ]
Sucção/Deglutição *(melhor resultado = 0 e pior = 22)*	[ ]	[ ]
Mastigação *(melhor resultado = 0 e pior = 13)*	___	[ ]
Deglutição semissólido/sólido *(melhor resultado = 0 e pior = 17)*	___	[ ]
Deglutição pastoso *(melhor resultado = 0 e pior = 22)*	[ ]	[ ]
Deglutição líquido *(melhor resultado = 0 e pior = 15)*	___	[ ]
**RESULTADO FUNÇÕES OROFACIAIS**	**[ ]**	**[ ]**
PONTO DE CORTE: FUNÇÕES OROFACIAIS	5	5
**ESCORE TOTAL OBTIDO (Exame Clínico Miofuncional Orofacial)**	**[ ]**	**[ ]**
PONTO DE CORTE: Distúrbio Miofuncional Orofacial (DMO)	9	14

Quanto aos domínios pertencentes aos encaminhamentos, seguem na Tabela 3 os valores encontrados a partir da análise dos pareceres emitidos pelo comitê de especialistas.

**Tabela 3:** Apresentação dos valores de acurácia diagnóstica, conforme valores de sensibilidade e especificidade, referente aos domínios de encaminhamentos para lactentes

Domínio	Faixa Etária (meses)	SE	ES	ACC	VP	FN	FP	VN	VPP	VPN
Encaminhamento para fonoaudiologia	6-11	13,3	84,0	57,5	2	13	4	21	33,3	61,8
	12-23	18,2	100	50,0	4	18	0	14	100	43,7
Outros encaminhamentos multidisciplinares	6-11	44,4	87,1	77,5	4	5	4	27	50	84,4
	12-23	50	78,6	61,1	11	11	3	11	78,6	50

**Legenda:** SE = Sensibilidade; ES = Especificidade; ACC = Acurácia; VP = Verdadeiro Positivo; FN = Falso Negativo; FP = Falso Positivo; VN = Verdadeiro Negativo; Maximizing Sensitivity with Minimum Specificity (Constrained Maximum Sensitivity); VPP = Valor Preditivo Positivo; VPN = Valor Preditivo Negativo

Com a função de resumir os achados encontrados nos resultados e discussão, segue a Tabela 4 relacionando o valor da acurácia e conceito para cada faixa etária de lactentes do protocolo MMBGR – Lactentes e Pré-escolares.

**Tabela 4:** Resumo dos valores de acurácia diagnóstica (%) e conceito, conforme domínios da avaliação miofuncional orofacial de lactentes e encaminhamentos, por faixa etária

Domínio	%Acurácia/conceito 06 a 11 meses	%Acurácia/conceito 12 a 23 meses
Estruturas orofaciais	45,0 - Não ideal	69,4 - Razoável
Tônus	70,0 – Razoável	66,7 - Razoável
Funções Orofaciais	42,5 - Não ideal	72,2 - Razoável
Motricidade Orofacial (DMO)	37,5 - Não ideal	88,9 – Ideal
Encaminhamento para Fonoaudiologia	57,5 - Não ideal	50,0 - Não ideal
Outros Encaminhamentos (Multidisciplinares)	77,5 – Razoável	61,1 - Razoável

**Legenda:** DMO = Distúrbio Miofuncional Orofacial

## DISCUSSÃO

O Protocolo de Avaliação Miofuncional Orofacial MMBGR – Lactentes e Pré-escolares é o primeiro instrumento avaliativo na área de MO a abranger faixa etária de seis a 71 meses de vida, que possibilita a atribuição de escores, com a finalidade de diagnosticar sujeitos com DMO. De acordo com as especificidades envolvidas, considerou-se apresentar os dados pertencentes exclusivamente à população de lactentes, incluindo o detalhamento por subgrupos conforme faixa etárias (Grupo 1: seis a 11 meses e 29 dias; Grupo 2: 12 a 23 meses e 29 dias).

A titulação e experiência dos avaliadores que compuseram o comitê de especialistas abrange da especialização em MO até o pós-doutoramento, com ampla expertise no atendimento clínico de lactentes na área de MO (a maioria de 87,5% com mais de cinco anos de tempo de atuação, sendo metade com mais de 16 anos), indica que as imagens foram analisadas por profissionais capacitados e com olhar diferenciado para esse público. A diversidade do comitê de especialistas atingiu quatro regiões da federação, sendo que na formação de trio para análise de cada participante, os avaliadores eram provenientes de diferentes estados, o que permitiu que a análise do instrumento abrangesse olhares e vivências distintas das variadas regiões do Brasil. O limite do estudo para atingir a região Sul, deu-se pelo declínio de alguns fonoaudiólogos, conforme já citado nos resultados.

Considera-se que o perfil do comitê de especialistas, que abrangeu vasta experiência docente e clínica na área de MO com lactentes, possibilitou que as análises das imagens fossem executadas com o rigor preconizado dos procedimentos desenhados para validação, o que aumenta o valor clínico relacionado ao conteúdo do instrumento em avaliar com eficácia e com capacidade para contribuir para o nível de cientificidade do trabalho^(4)^.

No que tange ao domínio de estruturas orofaciais do Protocolo de Avaliação Miofuncional Orofacial MMBGR - Lactentes e Pré-escolares, foram obtidos para lactentes resultados classificados como ideal (83,3%) e razoável (78,6%) para sensibilidade, sendo o instrumento capaz de identificar sujeitos com alteração nessas estruturas, quando alteradas. Já especificidade é considerada não ideal (28,6% e 37,5%), o que indica que o teste é pouco específico para predizer lactentes sem alteração na ausência delas.

A acurácia diagnóstica para o domínio de estruturas orofaciais foi considerada não ideal para o primeiro grupo etário e razoável para lactentes acima de 12 meses, evidenciando que o instrumento é capaz de detectar os sujeitos preditos com alteração de modo suficiente em lactentes mais velhos. Já, no grupo etário de seis a 11 meses, a proporção de VPN tiveram maior evidência do que na faixa etária de 12 a 23 meses. Isso mostra que para o primeiro grupo etário o instrumento consegue identificar melhor os lactentes sem alteração.

De fato, a necessidade de ajustes na posição e postura dos lactentes durante a obtenção da imagem pode ter sido um fator limitador, e portanto, ter influenciado na capacidade do instrumento identificar os lactentes preditos com alteração. O registro da avaliação em tempo real merece ser considerado para faixa etária mais jovem^(4)^. Por outro lado, pondera-se sobre a possibilidade de ampliar a indicação de exames complementares, tal como a ultrassonografia, para compor a avaliação e diagnóstico miofuncional orofacial em lactentes.

Quanto aos resultados que indicam que o domínio de estruturas orofaciais é mais acurado na avaliação de indivíduos acima de 12 meses do que na faixa etária de seis a 11 meses, considera-se que a realização de algum ajuste na posição e postura de lactentes na obtenção da imagem possa ser um recurso a ser utilizado, além da realização dos registros no protocolo na situação presencial.

Para o domínio tônus, os níveis de sensibilidade tiveram porcentagem maior de 60% para as duas faixas etárias, sendo considerado razoável, com indicação que o instrumento é suficiente na detecção de lactentes com alterações. A especificidade razoável para a primeira faixa etária (71%) e não ideal acima de 12 meses, significa que para identificação de sujeitos sem alteração na ausência delas, o primeiro grupo teve valores mais suficientes. Considera-se que a avaliação obtida por meio de análise de vídeo no presente estudo possa ter prejudicado o diagnóstico de alterações do tônus, visto que o avaliador é privado da informação proprioceptiva. O método de análise de imagens de vídeo é limitador para esse domínio, assim, recomenda-se que na clínica seja seguida a orientação dos autores do instrumento em recorrer à palpação no momento da avaliação^(5,6)^. O registro em tempo real do tônus poderia minimizar possíveis dificuldades de análise. Apesar disso, a acurácia diagnóstica no domínio tônus manteve-se razoável para todas as faixas etárias, logo, o instrumento teve valores considerados suficientes na exatidão de identificar o que se propôs a avaliar para tônus.

As funções orofaciais tiveram sensibilidades consideradas ideais (83,3% e 96,3%) para todas as faixas etárias de lactentes, logo, o instrumento é eficaz na identificação de sujeitos com alteração quando presentes. Já a especificidade é considerada não ideal para as funções orofaciais, sendo pouco eficaz na identificação de sujeitos sem alteração na ausência delas. Para a faixa etária de seis a 11 meses, os sujeitos preditos sem alteração em funções orofaciais foram melhor identificados (VPN de 92,3%) do que aqueles preditos com alteração.

A acurácia diagnóstica no domínio funções orofaciais manteve-se razoável somente para lactentes mais velhos. Isso poderia ser explicado pelo processo de amadurecimento fisiológico dos órgãos fonoarticulatórios desde o nascimento, cuja as alterações e padrões “atípicos” ficam mais evidentes com o avanço da idade. As diferenças são menos sutis com o avanço na estruturação e desenvolvimento da musculatura, logo, as adaptações que poderiam ter sido toleradas na fase de lactentes mais novos já devem dar lugar à execução de modo padrão^(20)^.

O domínio referente à alteração de MO diz respeito à presença ou não de DMO nos lactentes. Foram obtidos valores de sensibilidade ideais (81,2% e 96,9%) e especificidade não ideal para todas as idades de lactentes, logo, o instrumento mostrou-se suficiente para identificar sujeitos com alterações e insuficiente para descartar os sem alterações.

O resultado para acurácia para DMO foi não ideal em lactentes de seis a 11 meses e ideal para 12 a 23 meses, com VPP maiores (91,2%) nessa última faixa etária. Os achados indicam que, mesmo com sensibilidade ideal, o primeiro grupo teve menor capacidade de predizer sujeitos com alteração, o que pode ser justificado, pois processos atípicos no desenvolvimento podem ser mais sutis em idades iniciais, enquanto que em idades posteriores, em razão das mudanças nas estruturas físicas, neurológicas, cognitivas e comportamentais estes processos podem ser mais evidenciados^(21)^.

Sobre os valores obtidos para os escores de ponto de corte de cada domínio específico do Protocolo MMBGR lactentes e pré-escolares, considera-se que mesmo valores baixos já são indicativos de alterações nos domínios avaliados^(22)^. Por exemplo, em relação às estruturas orofaciais o instrumento prevê a atribuição de escores de 0 (melhor resultado) até 62 (pior resultado) e a nota de corte ficou 7 para G1 e 8 para G2. Da mesma forma, isso ocorre para tônus que prevê pontuação entre 0 e 6, e a obtenção de 1 ponto já aponta para alteração, e para funções orofaciais que prevê resultados entre 0 e 46 para G1 e entre 0 e 92 para G2, e teve o ponto de corte de 5 como indicativo de alteração. Assim, pontuações baixas nos domínios específicos já devem requerer atenção especial do clínico quanto aos aspectos que envolvem alterações miofuncional orofacial em lactentes.

Da mesma forma como ocorreu nos domínios específicos, quanto aos escores totais para DMO, os valores de 9 (lactentes entre 6 meses a 11 meses e 29 dias) e de 14 (lactentes entre 12 meses a 23 meses e 29 dias), já apontam para a importância do olhar do clínico para o DMO e respectiva necessidade de acompanhamento fonoaudiológico e/ou multidisciplinares. A avaliação pode fornecer subsídios para reabilitação e encaminhamentos em idades iniciais da vida, cruciais quanto ao crescimento e desenvolvimento do sistema estomatognático^(23)^ e a falta de avaliação e/ou intervenção fonoaudiológica em tempo oportuno pode implicar em consequências até a vida adulta^(9)^.

As alterações identificadas no primeiro ano de vida apresentam maior probabilidade de resolução^(24)^. Além disso, dada a relação da Fonoaudiologia com as demais áreas da saúde, os profissionais devem ser capazes de indicar os acompanhamentos multidisciplinares^(10)^. Entretanto, apesar das necessidades (ou não) de acompanhamento fonoaudiológico e/ou encaminhamentos a outros profissionais terem sido tratadas como domínios para cálculos de acurácia diagnóstica, o ponto central do protocolo é que sejam registrados aspectos inerentes à avaliação oromiofuncional, o que inclui estruturas (estáveis e móveis), tônus e respectivas funções orofaciais com atribuições de escores.

A necessidade de encaminhamento para Fonoaudiologia foi um domínio avaliado, devido a sua importância para reabilitação de indivíduos preditos com alteração. Nos dois grupos de lactentes, destacou-se ser maior a especificidade (84,0% e 100%) do que a sensibilidade, evidenciando que indivíduos sem necessidade de encaminhamento foram melhor identificados do que aqueles com necessidade, principalmente em idades dos seis aos 11 meses (VPP de 61,8%). Para ambos os grupos de lactentes, a acurácia diagnóstica sobre a indicação de acompanhamento fonoaudiológico para lactentes foi classificada como não ideal. Pondera-se em idades mais avançadas as possíveis adaptações miofuncionais orofaciais sejam menos toleradas pelo clínico, existindo maior ocorrência dos encaminhamentos. Além disso, assim como em outros domínios anteriormente discutidos, esse dado pode estar relacionado à natureza do estudo do tipo validação, cujas análises foram realizadas a partir dos registros gravados de imagens e não da situação da avaliação em tempo real.

O último domínio avaliado, sobre a necessidade de outros encaminhamentos multidisciplinares (Odontologia, Otorrinolaringologia, entre outros), apresentou valores de sensibilidade e especificidade similares ao domínio relacionado ao encaminhamento para Fonoaudiologia. Apesar de ambos os grupos de lactentes terem valores de sensibilidade não ideal, na faixa etária de seis a 11 meses o VPN foi ideal (84,4%) o que é considerado importante para não serem realizados mais encaminhamentos do que aqueles realmente necessários em uma etapa de vida que já requer tantos cuidados gerais e específicos de puericultura. Em lactentes acima de 12 meses o VPP é considerado razoável (78,6%) para predizer a necessidade do encaminhamento multidisciplinar, o que é fundamental no direcionamento do tratamento, sendo que a ausência desse encaminhamento pode representar uma situação de risco na presença de um DMO não diagnosticado e tratado^(25)^. Ressalta-se a que a acurácia diagnóstica manteve-se razoável para todas as faixas etárias, sobretudo devido à natureza dos itens analisados, com predominância das imagens estáticas (Odontologia: dentes e oclusão; Otorrinolaringologia: frênulo de língua, tonsilas palatinas e marcação do fluxo nasal com uso do espelho).

Apesar das dificuldades de recrutamento do comitê de fonoaudiólogos especialistas, todas as atividades foram cumpridas na totalidade, a fim de alcançar o objetivo de determinar o ponto de corte para DMO. A principal limitação do estudo refere-se à natureza da metodologia de análise dos dados dos estudos de validação, que prevê a análise dos casos a partir do registro das imagens gravadas (estáticas e dinâmicas), o que nem sempre é ideal para avaliação de determinado domínio analisado. Pondera-se que o fonoaudiólogo ao utilizar o Protocolo MMBGR - Lactentes e Pré-escolares na rotina da clínica fonoaudiológica possa fazer uso do protocolo com registro em tempo real, além de checar posteriormente as imagens obtidas junto ao lactente, o que possivelmente auxiliará na acurácia ideal dos itens/domínios que compõem o diagnóstico do DMO.

As limitações de instrumentos de diagnóstico do DMO poderiam ser reduzidas com a ampliação de protocolos que sejam capazes de avaliar a MO em idades iniciais da vida, com a ressalva de que ainda é restrito o número de instrumentos voltados para essa faixa etária. Como perspectiva futura, pondera-se que o uso de outros parâmetros observáveis para compor a avaliação miofuncional orofacial em lactentes, com o uso de exames instrumentais, como de ultrassonografia^(26,27)^, possa vir a ser uma alternativa interessante para complementar o diagnóstico do DMO. Destaca-se, entretanto, que o reconhecimento de indivíduos com alteração continua a ser o principal objetivo do Protocolo MMBGR – Lactentes e Pré-escolares.

## CONCLUSÃO

O Protocolo de Avaliação Miofuncional Orofacial MMBGR – Lactentes e Pré-escolares é um recurso para uso na clínica e na pesquisa fonoaudiológica, no estabelecimento de diagnóstico em idades iniciais da vida. A atribuição de pontos de corte possibilita que o fonoaudiólogo reconheça o DMO, utilize como parâmetro para reavaliações, contribuindo para acompanhamento longitudinal do paciente.

O Protocolo de Avaliação Miofuncional Orofacial MMBGR – Lactentes e Pré-escolares, quando analisado por meio de fotos e filmagens é acurado para lactentes entre seis a 11 meses somente quanto ao tônus; e para lactentes de 12 a 23 meses em todos domínios do exame, sendo ideal para diagnosticar DMO. O instrumento é razoável quanto à necessidade de encaminhamentos multidisciplinares para toda faixa etária de lactentes.

O estudo sobre os pontos de corte no Protocolo MMBGR - Lactentes e Pré-escolares é um marco para a clínica e pesquisa fonoaudiológica, visto que este é um importante instrumento no Brasil na área de MO. Além disso, a presente pesquisa pode contribuir como guia para novas pesquisas que envolvam a avaliação miofuncional orofacial em idades iniciais de vida.
